# Point-of-care ultrasound in Management for Dyspneic Uremic Patients: a case report

**DOI:** 10.1186/s12882-019-1654-x

**Published:** 2019-12-12

**Authors:** Pei-Hsiu Wang, Wan-Ching Lien

**Affiliations:** Department of Emergency Medicine, National Taiwan University Hospital and College of Medicine, National Taiwan University, |No.7, Chung-Shan South Road, Taipei, 100 Taiwan

**Keywords:** Point-of-care ultrasound, Uremia, Uremic pericarditis, Pericardial effusion

## Abstract

**Background:**

Point-of-Care Ultrasound (PoCUS) is considered as an extension of clinicians’ patient care and can be integrated into daily clinical practice. Dyspnea is a common presentation in uremic patients. With the aids of PoCUS and integrated assessments of lung, heart and inferior vena cava (IVC), the etiology of dyspnea in uremic patients can be determined earlier.

**Case presentation:**

A 67-year-old woman presented with progressive shortness of breath and bilateral legs edema for 3 weeks. The laboratory data revealed marked elevated level of serum creatinine and blood urea. A large amount of pericardial effusion was timely detected by PoCUS. Uremic pericarditis was suspected. Emergent hemodialysis was initiated and her symptoms improved.

**Conclusions:**

PoCUS is a noninvasive and cost-effective imaging modality and it has been popular in the emergency department (ED). In uremic patients presenting with dyspnea, the integration of PoCUS into traditional physical examinations help emergency physicians narrow down the differential diagnoses.

## Background

Point-of-care Ultrasound (PoCUS) is increasingly used to address specific questions in daily clinical practice. PoCUS has broad-spectrum applications as an extension of patient care, beside physical examinations and laboratory data [1].

Dyspnea is a common presentation in patients with uremia. Nowadays, uremic pericarditis is rare but life-threatening because of the complication of cardiac tamponade. Delayed diagnosis and management are associated with high mortality and morbidity [2]. Therefore, timely recognition is essential. We present a dyspneic uremic patient in whom a large amount of pericardial effusion was timely detected by PoCUS. Uremic pericarditis was suspected and emergent hemodialysis was initiated.

## Case presentation

A 67-year-old woman presented with progressive shortness of breath and bilateral legs edema for 3 weeks. In addition, generalized pruritus and poor appetite were noted. She reported no obvious decrease in urine output. Her medical history included untreated diabetes mellitus and chronic kidney disease (CKD). She denied use of Chinese herbs and pain killers recently. She denied fever, chest pain, abdominal pain or tarry stool.

**Fig. 1:**
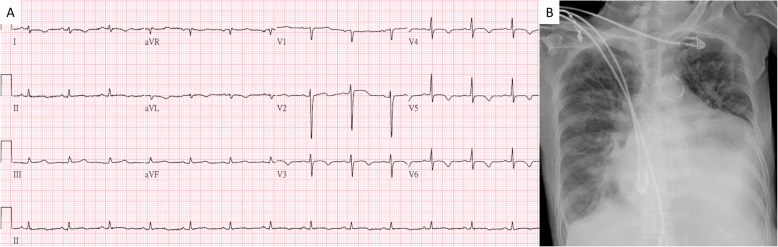
Electrocardiogram and chest x-ray. **a** Electrocardiogram shows normal sinus rhythm with low voltage QRS. **b** Chest x-ray shows enlargement of the cardiac silhouette

**Fig. 2:**
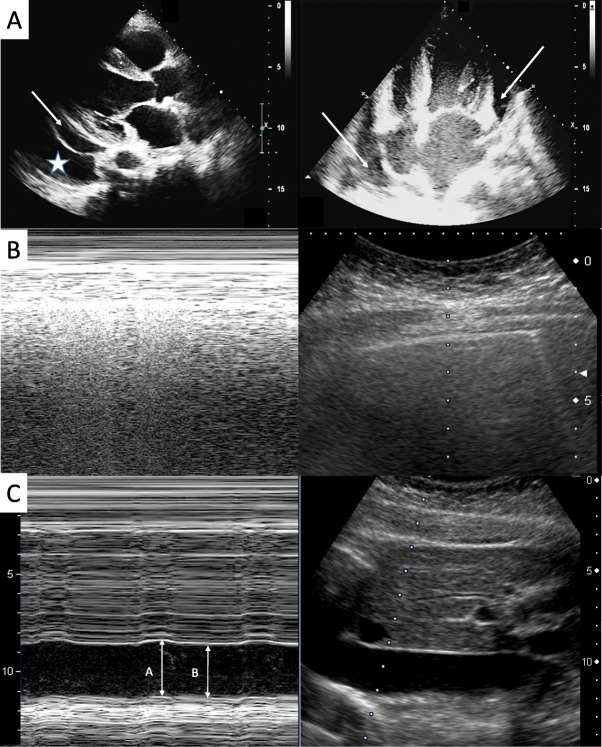
Point-of-care ultrasound. Machine: Toshiba Aplio MX SSA-780A. Probe: micro convex (cardiac ultrasound), convex (lung and IVC ultrasound) **a** Focus cardiac ultrasound with long-axis view and four chamber view. Large amount of pericardial effusion (arrows). Pleural effusion (star). **b** Lung ultrasound. Normal lung sliding without prominent B lines. Normal lung pattern on M-mode: the seashore sign. **c** IVC ultrasound. The IVC diameter was measured as A (3 cm, during expiration) and B (2.7 cm, during inspiration). M-mode showed minimal change during respiratory cycle.

Upon arrival to the ED, she was oriented and her vital signs were as followings: a heart rate of 83 bpm, blood pressure of 198/91 mmHg, respiratory rate of 18, body temperature of 37 °C and oxygen saturation of 97% in room air. Chest auscultation revealed crackles at both bases without heart murmurs or friction rubs. Pitting edema over bilateral lower limbs up to the knees was noted. Physical examination was otherwise unremarkable. The laboratory data revealed marked elevated level of serum creatinine (548 μmol/L) and blood urea (35 mmol/L). Urine analysis revealed presence of proteinuria (3+ at the dipstick) but serum albumin (32 g/L) was not obviously reduced. Arterial blood gas showed metabolic acidosis. Electrocardiogram (ECG) showed normal sinus rhythm with low voltage QRS (Fig. 1a), and chest X-ray revealed an enlargement of the cardiac silhouette (Fig. 1b). PoCUS was applied and included assessments of lung, heart and inferior vena cava (IVC). Sonography disclosed a large amount of pericardial effusion without right ventricle collapse sign (Fig. 2a), normal lung sliding signs with one B line (Fig. 2b) and mildly distended IVC (Fig. 2c).

Uremic pericarditis was highly suspected. Intensive daily hemodialysis was initiated and the amount of fluid removal was 1 kg per day. She tolerated dialysis well and her symptoms improved after five days. Daily hemodialysis was then shifted to standard hemodialysis (three times a week). A follow-up echocardiography showed resolution of pericardial effusion. The patient was discharged in a stable condition.

## Discussion and conclusions

**Fig. 3:**
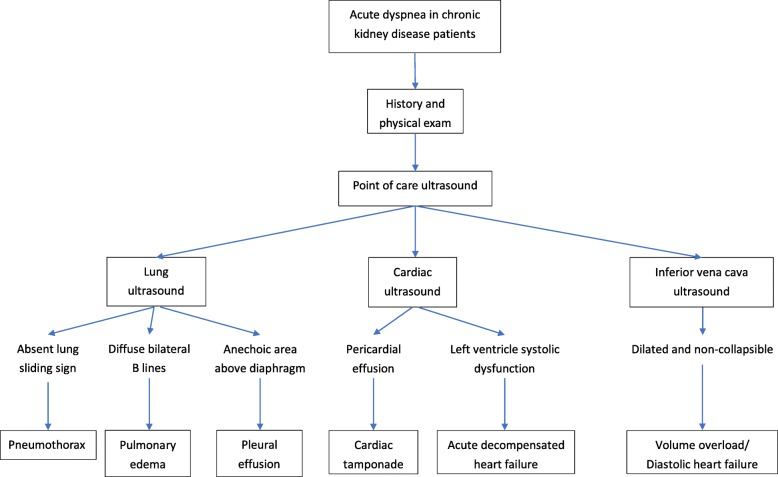
Algorithm for the evaluation of chronic kidney disease patients with acute dyspnea

Traditionally, physicians use inspection, palpation and then stethoscopes to evaluate patients. In the recent decades, ultrasound has been applied as a “21th century stethoscope” to help physicians better visualize internal organs and detect various diseases [3]. PoCUS is a multi-organ evaluation and can be integrated into traditional physical examinations [4]. There are an increasing number of structured PoCUS protocols for clinical scenarios, such as RUSH for shock, [5] FEEL [6] and US-CAB [7] for resuscitation. According to the applications and skills of POCUS in chronic kidney disease (CKD) patients described in previous literature, we developed a diagnostic algorithm integrating PoCUS for the evaluation of dyspnea in the CKD patient (Fig. 3) [8, 9].

Dyspnea is a frequent manifestation in emergency department (ED) patients, especially with uremia. However, it can result from many conditions. Early recognition, diagnosis and proper management are mainstays to reduce morbidity and mortality. Lung ultrasound exhibits high diagnostic accuracy for pleural effusion, lung consolidations, interstitial syndrome, and pneumothorax [10]. Signs of left ventricular systolic dysfunction, lung congestion, right ventricular enlargement, and elevated central venous pressures are often missed by physical techniques, but can be easily detected by PoCUS [4]. In this case, we integrated lung, heart and IVC ultrasound, as an adjunct to clinical examinations, to evaluate the patient’s dyspnea. A few B lines indicated his dyspnea was not related to pulmonary edema. The patient’s dyspnea was mainly caused by massive pericardial effusion. Uremic pericardial effusion is thought to result from the accumulation of toxic metabolites. The inflammation of pericardium causes the chronic reduction of pericardial compliance and the slow rise in intrapericardial pressure. The most important treatment is the initiation of dialysis [2].

There are two kinds of end-stage renal disease (ESRD)-related pericardial diseases: uremic pericarditis and dialysis-associated pericarditis. Uremic pericarditis occurs in the non-dialysis patients with untreated uremia. Dialysis pericarditis occurs in chronic-dialysis patients with inadequate dialysis or fluid overload [2]. Early diagnosis for uremic pericarditis is challenging for clinicians currently because of the low incidence caused by the widespread availability of dialysis. Besides, the typical presentations of pericarditis such as fever, pleuritic chest pain, friction rub could not occur in all patients. Moreover, the characteristic finding of diffuse ST elevation in ECG is rare in uremic pericarditis because the myocardium is not involved [11]. Therefore, PoCUS can play an important role to rule in or rule out pericardial effusion in clinical practice.

Some limitations would exist in diagnostic ultrasound in patients with multi-diagnoses and multi-comorbidities. For example, patients with congestive heart failure and pneumonia would have pleural effusion, prominent B lines and lung consolidation. However, with gathering useful clinical information, ultrasonographers could overcome this problem based on the clinical situation of the patients [12]. In uremic patients presenting with dyspnea, the integration of PoCUS into traditional physical examinations help emergency physicians narrow down the differential diagnoses.

## Data Availability

Not applicable.

## Abbreviations

CKD: Chronic kidney disease
ECG: Electrocardiogram
ED: Emergency department
ESRD: End-stage renal disease
IVC: Inferior vena cava
PoCUS: Point-of-Care Ultrasound
